# 

**Published:** 1910-11

**Authors:** 


					﻿PUBLISHED MONTHLY.
SUBSCRIPTION $1.0P
ATLANTA
JOURNAL-REGD13®
OF MEDICING' m
S Atlanta Medical and Surgical Journal, Establlstefl1355. ytTiu
and Southern Medical Record, Established 1870.	) 3Dili TvOI
Vol. L/VT.	Atlanta, Ga., November, 1910.	No. 8
				

